# Blood Pressure Changes During Mechanical Thrombectomy for Acute Ischemic Stroke Are Associated With Serious Early Treatment Complications: Symptomatic Intracerebral Hemorrhage and Malignant Brain Edema

**DOI:** 10.3389/fneur.2022.884519

**Published:** 2022-07-05

**Authors:** Marcin Wiącek, Maciej Szymański, Klaudia Walewska, Halina Bartosik-Psujek

**Affiliations:** Institute of Medical Sciences, University of Rzeszow, Rzeszów, Poland

**Keywords:** stroke, thrombectomy, blood pressure, intracranial hemorrhage, cerebral edema

## Abstract

**Background:**

Symptomatic intracranial hemorrhage (sICH) and malignant brain edema (MBE) are well-known deleterious endovascular treatment (EVT) complications that some studies found to be associated with postprocedural blood pressure (BP) variability. We aimed to evaluate their association with periprocedural BP changes, including its intraprocedural decrease.

**Methods:**

We retrospectively analyzed the data of 132 consecutive patients that underwent EVT between 1 December 2018 and 31 December 2019, for anterior circulation ischemic stroke. Analyzed predictors of sICH and MBE included non-invasively obtained BP before and 5-min after treatment, intraprocedural relative decreases of BP from baseline, and its post-treatment increases. SICH was defined in accordance with the Safe Implementation of Thrombolysis in Stroke-Monitoring Study (SITS-MOST) criteria and MBE as brain edema with midline shift on the follow-up imaging. We used binary logistic regression analysis to investigate the association of BP parameters and the incidence of sICH and MBE.

**Results:**

Among the included patients, 11 (8.3%) developed sICH and 31 (23.5%) MBE. The intraprocedural decrease of mean arterial blood pressure (MAP) was independently associated with MBE occurrence (aOR per 10 mmHg drop from baseline 1.27; 95% CI 1.01–1.60; P = 0.040). Over 40% MAP drop was associated with a higher risk of sICH in the entire cohort (aOR 4.24; 95% CI 1.33–13.51; P = 0.015), but not in the subgroup with successful reperfusion (aOR 2.81; 95% CI 0.64–12.23; P = 0.169). Post-treatment systolic blood pressure (SBP) and MAP elevation above their minimal values during MT are significantly associated with the development of sICH (aOR per 10 mmHg SBP increase 1.78; 95% CI 1.15–2.76; P = 0.010 and aOR per 10 mmHg MAP increase 1.78; 95% CI 1.04–3.03; P = 0.035).

**Conclusions:**

In the anterior circulation ischemic stroke patients relative MAP decrease during EVT is associated with a higher risk of MBE occurrence, and over 40% MAP drop with a higher incidence of both MBE and sICH. Post-treatment elevation of SBP and MAP increased the risk of sICH.

## Introduction

Significant oscillations of periprocedural blood pressure (BP) in patients with acute ischemic stroke (AIS) treated with mechanical thrombectomy (MT) are now considered to be a common finding (1). Accumulating evidence indicates that they may influence treatment outcomes. Higher BP before endovascular treatment (EVT), BP decreases during MT, its high post-treatment values, and periprocedural variability were all found to be associated with poor functional outcomes (2–4). However, the underlying mechanisms of this detrimental impact on the long-term prognosis are not well understood.

It is hypothesized that blood pressure decreases in the setting of large vessel occlusion (LVO) compromises collateral flow and promotes the progression of penumbra to the ischemic core, as well as exacerbates endothelial ischemic injury. After reperfusion treatment with complete or only partial LVO recanalization, BP elevation may result in hemorrhagic transformation (HT) of AIS or reperfusion-induced cerebral edema (CED) (5). Findings of final infarct volume (FIV) increment with BP decreases during MT and higher sICH rates with its postprocedural higher values may confirm those assumptions (6, 7). Considering the pathophysiology of hemorrhagic and edematous complications after reperfusion therapy, it is highly probable that periprocedural BP oscillations may promote the occurrence of HT and CED, including their most devastating forms: symptomatic intracranial hemorrhage (sICH) and malignant brain edema (MBE). To our knowledge, no data about the association of BP decrease during MT with those serious EVT complications has been presented.

SICH and MBE are known to have serious deleterious consequences on MT-treated patients with AIS and efforts should be made to find their modifiable risk factors. In this study, we examined the effects of periprocedural BP changes on those early EVT complications.

## Materials and Methods

### Study Design

We conducted a retrospective observational study of consecutive patients with AIS and LVO undergoing EVT. The study was conducted at the academic comprehensive stroke center: Department of Neurology, Clinical Regional Hospital No. 2, Rzeszow, Poland. It was confirmed by the Bioethics Committee of the University of Rzeszow. Written consent was waived due to its observational status.

### Subjects

We retrospectively analyzed the prospective database comprising adult (age ≥ 18 years) patients with AIS due to occlusion of the intracranial internal carotid artery (ICA) and/or middle cerebral artery (MCA) in its first (M1) or second (M2) segment. All patients were treated with EVT using modern stent retrievers and/or direct aspiration technique over a period of 13 months (1 December 2018 and 31 December 2019). Patient demographic data, medical history, baseline characteristics, and imaging data were obtained from electronic medical records.

All treatment decisions were made by the endovascular treatment team (experienced vascular neurologist and neuroradiologist) in accordance with the Polish Neurological Society stroke treatment guidelines (8). The choice of anesthesia type (general anesthesia or conscious sedation) in each case was a collegial decision of the anesthesiologist, neurologist, and neuroradiologist. There were no specific recommendations regarding periprocedural BP levels. Hypotension was treated with ephedrine, phenylephrine, or norepinephrine at the anesthesia treatment team's discretion.

### Patients and Treatment Parameters

Periprocedural variables included age, sex, comorbidities, admission NIHSS, onset-to-groin puncture time (minutes), onset-to-reperfusion time (minutes), procedure duration (minutes), localization of LVO, type of anesthesia, administration of bridging intravenous thrombolysis (IVT), and additional procedures performed during hospitalization: intra-/extracranial stenting and/or decompressive hemicraniectomy.

Post-MT reperfusion was graded by the Thrombolysis in Cerebral Infarction (TICI) scale: grades 2b (perfusion >50% of the vascular distribution of the occluded artery) and 3 (full perfusion with the filling of all distal branches) were considered successful reperfusion (9).

Functional status and mortality at 3-months (using the modified Rankin Scale, mRS) were evaluated either in person or by telephone in accordance with our standard procedure.

### Blood Pressure Data

Blood pressure data were obtained from the patient's anesthesia report following the intervention. These data included systolic blood pressure (SBP), diastolic blood pressure (DBP), and mean blood pressure (MAP) measured non-invasively before EVT procedure, every 5 min during MT, and 5 min after procedure termination (postprocedural BP).

Baseline BP was defined as the value of the measurement directly preceding induction of anesthesia. The lowest intraprocedural BP measured before successful reperfusion or termination of the procedure in case of unsuccessful reperfusion was considered as the intraprocedural minimal BP value and the difference between baseline and minimal BP as the maximal BP drop.

### Outcome Measurements

All patients underwent non-contrast CT or magnetic resonance imaging as part of their routine clinical work-up at 24–36 h post-EVT or earlier/later if significant clinical deterioration occurred. One patient was excluded from the study due to death before having an imaging study performed. The imaging characteristics were assessed by two experienced neurologists. If discrepancies occurred, the final result was determined by consensus opinion.

SICH was defined in accordance with Safe Implementation of Thrombolysis in Stroke-Monitoring Study (SITS-MOST) criteria as parenchymal hematoma (PH) type 2 (hematoma occupying >30% of the infarcted area with significant space-occupying effect) with the National Institutes of Health Stroke Scale (NIHSS) score increment ≥4 points (10). CED subtypes were defined as follows: CED-0 as no brain edema, CED-1 as focal brain swelling limited to less than one-third of the hemisphere, CED-2 as brain swelling of more than one-third of the hemisphere, and CED-3 as brain edema causing midline shift. Referring to previous studies, MBE was defined as CED-3 (11).

### Statistical Analyses

Analyzed variables were presented as mean ± standard deviation (SD) or median (interquartile range) depending on the normality of the distribution. Categorical variables were presented as numbers (percentage). Univariate analyses were performed using chi-square or Fisher's exact test for dichotomous variables and 2-tailed *t*-test or Mann-Whitney U-test for continuous variables, as appropriate. Associations of hemodynamic variables with outcome parameters were determined using ordinal logistic regression models adjusted for predefined factors selected according to prior studies and theoretical considerations. Analyses of sICH associations were adjusted for recanalization status, bridging thrombolysis, NIHSS score at admission, onset-to-reperfusion time, and MBE for recanalization status, NIHSS score at admission, onset-to-reperfusion time, presence of intracranial hemorrhage on post-treatment brain imaging. Subgroup analysis of patients with successful recanalization was further performed (adjusted for the same variables apart from recanalization status). All statistics were computed using PQStat Software 1.8 (Poznan, Poland). Statistical significance was set at P < 0.05 (two-tailed).

## Results

One hundred thirty-two patients were included with a mean age of 70 ± 11 years, 67 (50.8%) females, and 15.4 ± 5.6 NIHSS score at admission. SICH was found in 11 (8.3%) and MBE in 31 (23.5%) cases. Six patients had an extracranial portion of ICA stenting and in 3 subjects decompressive hemicraniectomy was performed. Almost all of the reviewed patients (97%) experienced MAP decrease during EVT. Baseline characteristics and periprocedural hemodynamic measures were divided into subgroups according to the outcome measures (ICH and MBE) and presented in Table 1.

**Table 1:** Univariate analyses of baseline characteristics according to symptomatic intracranial hemorrhage and malignant brain edema.

	**sICH**			**MBE**		
	**Yes (*n* = 11)**	**No (*n* = 121)**	***p*-value**	**Yes (*n* = 31)**	**No (*n* = 101)**	***p*-value**
Age, years (mean ± SD)	74.1 ± 12.5	69.6 ± 11.0	0.205	72.7 ± 9.9	69.2 ± 11.4	0.119
Female, *N* (%)	6 (54.5)	61 (50.4)	0.793	18 (58.1)	49 (48.5)	0.352
**Medical history**						
Hypertension, *N* (%)	10 (90.1)	92 (76.0)	0.45	28 (90.3)	74 (73.3)	0.047
Hyperlipidemia, *N* (%)	1 (9)	25 (20.7)	0.692	4 (12.9)	22 (21.8)	0.278
Atrial fibrillation, *N* (%)	7 (63.6)	74 (61.2)	1.0	15 (48.4)	66 (65.4)	0.090
Diabetes mellitus, *N* (%)	1 (9)	24 (19.8)	0.690	5 (16.1)	20 (19.8)	0.648
CAD, *N* (%)	2 (18.2)	27 (22.3)	1.0	5 (16.1)	24 (23.8)	0.369
**Baseline characteristics**						
Baseline NIHSS (mean ± SD)	17.6 ± 5.8	15.2 ± 5.6	0.177	18.0 ± 4.3	14.7 ± 5.8	0.004
Onset-to-groin time, minutes (median, IQR)	245 (227–315)	267 (218–315)	0.967	270 (245–319)	265 (210–315)	0.265
Onset-to-reperfusion time, minutes (median, IQR)	311 (288–439)	350 (280–405)	0.811	375 (311–419)	348 (275–400)	0.097
Procedure duration, minutes (median, IQR)	87 (58–164)	75 (50–114)	0.392	88 (57–143)	75 (48–109)	0.112
Bridging thrombolysis, *n* (%)	8 (72.7)	85 (70.2)	1.0	21 (67.7)	72 (71.3)	0.705
**Localization of occlusion**, ***n*** **(%)**						
ICA	7 (63.6)	24 (19.8)	0.004	15 (48.4)	16 (15.8)	<0.001
MCA M1	4 (36.4)	74 (61.2)	0.123	13 (41.9)	65 (64.4)	0.026
MCA M2	0 (0)	23 (19.0)	0.211	3 (9.7)	20 (19.8)	0.194
General anesthesia, *n* (%)	11 (100)	97 (80.1)	0.214	28 (90.3)	80 (79.2)	0.160
**Clinical and imaging treatment outcome measures**						
Successful recanalization, *n* (%)	8 (72.7)	98 (81.0)	0.452	21 (67.8)	85 (84.1)	0.044
ICH*, n* (%)	-	-	-	22 (70.9)	25 (24.8)	<0.001
Parenchymal hematoma type-2, *n* (%)	-	-	-	13 (41.9)	5 (5.0)	<0.001
3 months mRS ≤ 2, *n* (%)	0 (0)	54 (44.7)	0.003	1 (3.2)	53 (52.5)	<0.001
In-hospital mortality, *n* (%)	9 (81.8)	16 (13.2)	<0.001	18 (58.1)	7 (6.9)	<0.001
3 months mortality, *n* (%)	9 (81.8)	20 (16.5)	<0.001	19 (61.3)	10 (9.9)	<0.001

*MBE, malignant brain edema; ICH, intracranial hemorrhage; sICH, symptomatic intracranial hemorrhage; CAD, coronary artery disease, SBP, systolic blood pressure; MAP, mean arterial pressure; MT, mechanical thrombectomy, mRS, modified Rankin scale; ICA, internal carotid artery; MCA, middle cerebral artery; NIHSS, National Institutes of Health Stroke Scale*.

Among patients with sICH the higher rate of ICA occlusion (63.6 vs. 19.8%, P = 0.004), >40% drop of MAP compared to baseline (63.6 vs. 28.1%, P = 0,035), and higher increase of postprocedural MAP (30.0 vs. 18.3, P = 0.022) and SBP (44.4 vs. 27.8, P = 0.038) over their minimal intraprocedural values were observed (Table 2). More than 40% MAP decrease (aOR 4.17; 95% CI 1.11–15.72; P = 0.034), and postprocedural MAP (aOR per 10 mmHg over minimal intraprocedural value 1.28; 95% CI 1.02–1.62; P = 0.037) and SBP (aOR per 10 mmHg over minimal intraprocedural value 1.56; 95% CI 1.04–2.32; P = 0.030) elevation were found to be independent predictors of sICH, as shown in the Table 3.

**Table 2:** Univariate analyses of hemodynamic variables according to symptomatic intracranial hemorrhage and malignant brain edema.

	**sICH**			**MBE**		
	**Yes (*n* = 11)**	**No (*n* = 121)**	***p*-value**	**Yes (*n* = 31)**	**No (*n* = 101)**	***p*-value**
Baseline SBP, mmHg (mean ± SD)	157.8 ± 32.8	150.5 ± 26.6	0.392	160.0 ± 29.7	148.4 ± 25.7	0.036
Baseline MAP, mmHg (mean ± SD)	110.9 ± 25.2	107.0 ± 17.4	0.497	113.4 ± 21.6	105.5 ± 16.6	0.033
Minimal SBP, mmHg (mean ± SD)	95.7 ± 17.7	102.2 ± 19.0	0.281	99.0 ± 18.7	102.4 ± 18.9	0.379
Minimal MAP, mmHg (mean ± SD)	69.5 ± 14.7	74.0 ± 14.3	0.321	71.6 ± 15.3	74.3 ± 14.0	0.366
Post-MT SBP, mmHg (mean ± SD)	140.1 ± 22.7	130.0 ± 21.3	0.136	136.4 ± 29.7	129.1 ± 18.2	0.102
Post-MT MAP, mmHg (mean ± SD)	99.6 ± 16.1	92.3 ± 13.3	0.093	94.1 ± 15.7	92.6 ± 13.0	0.586
MAP maximal drop, mmHg (mean ± SD)	41.2 ± 22.1	33.0 ± 20.8	0.214	41.7 ± 20.9	31.2 ± 20.4	0.014
MAP maximal drop, % (mean ± SD)	35.5 ± 14.5	29.4 ± 15.7	0.221	35.6 ± 13.9	28.2 ± 15.8	0.020
>10% MAP drop, *n* (%)	11 (100)	104 (86.0)	0.358	30 (96.8)	85 (84.1)	0.074
>20% MAP drop, *n* (%)	8 (72.7)	84 (69.4)	1.0	26 (83.9)	66 (65.3)	0.049
>40% MAP drop, *n* (%)	7 (63.6)	34 (28.1)	0.035	17 (54.8)	24 (23.8)	0.001
Post-MT SBP elevation above minimal SBP, mmHg (mean ± SD)	44.4 ± 26.1	27.8 ± 24.5	0.038	37.4 ± 33.0	26.7 ± 21.5	0.037
Post-MT MAP elevation above minimal MAP, mmHg (mean ± SD)	30.0 ± 19.7	18.3 ± 15.7	0.022	22.5 ± 19.3	18.3 ± 15.2	0.211

*MBE, malignant brain edema; sICH, symptomatic intracranial hemorrhage; SBP, systolic blood pressure; MAP, mean arterial pressure; MT, mechanical thrombectomy*.

**Table 3:** Multivariate logistic regression analyses for symptomatic intracranial hemorrhage and *malignant cerebral edema*.

	**sICH**			**MBE**		
	**OR**	**CI 95%**	***p*-value**	**OR**	**CI 95%**	***p*-value**
Baseline SBP [10 mmHg]	1.08	0.86–1.35	0.517	1.19	0.99–1.42	0.062
Baseline MAP [10 mmHg]	1.07	0.76–1.50	0.707	1.23	0.96–1.58	0.109
MAP maximal drop [10 mmHg]	1.16	0.86–1.56	0.335	1.27	1.01–1.60	**0.040**
MAP maximal drop [10%]	1.24	0.80–1.92	0.335	1.42	1.03–1.97	**0.034**
>20% MAP drop	1.04	0.26–4.27	0.951	2.83	0.88–9.14	0.082
>40% MAP drop	4.17	1.11–15.72	**0.034**	4.28	1.57–11.66	**0.004**
Post-MT SBP elevation above minimal SBP [10 mmHg]	1.28	1.02–1.62	**0.037**	1.22	1.01–1.47	**0.035**
Post-MT MAP elevation above minimal MAP [10 mmHg]	1.56	1.04–2.32	**0.030**	1.21	0.91–1.60	0.186

*Bold font indicates statistical significance. An analysis of each parameter association with sICH, the multivariate logistic regression model was adjusted for recanalization status, bridging thrombolysis, NIHSS at admission, and onset-to-reperfusion time. An analysis of each parameter association with COED-3 the multivariate logistic regression model was adjusted for recanalization status, NIHSS at admission, onset-to-reperfusion time, and presence of intracranial hemorrhage on post-treatment brain imaging. sICH, symptomatic intracranial hemorrhage; MBE, malignant brain edema; SBP, systolic blood pressure; MAP, mean arterial pressure; MT, mechanical thrombectomy*.

In our cohort, the occurrence of MBE was significantly associated with the history of hypertension (P = 0.047), baseline NIHSS (P = 0.004), and intracranial ICA occlusion (P < 0.001). Periprocedural hemodynamic parameters associated with MBE were: higher baseline SBP (P = 0.036) and MAP (P = 0.033), intraprocedural maximal MAP decrease compared to baseline value calculated as the absolute value (P = 0.014), and a fraction of baseline MAP (P = 0.020), over 20% (P.049), >40% (P = 0.001) intraprocedural MAP decrease, and postprocedural SBP elevation (P = 0.037). After adjusting for potential confounders, the independent MBE predictors were: intraprocedural MAP decrease (aOR per 10 mmHg drop 1.27; 95% CI 1.01–1.60; P = 0.040, aOR per 10% fraction of baseline value drop 1.42; 95% CI 1.03–1.97; P = 0.040, aOR for over 40% drop 4.28; 95% CI 1.57–11.66; P = 0.004) and postprocedural SBP elevation (aOR per 10 mmHg 1.22; 95% CI 1.01–1.47; P.035).

In the subgroup analysis of patients with successful recanalization, >40% intraprocedural MAP drop was the only hemodynamic variable found to be independently associated with the risk of MBE (aOR 4.24; 95% CI 1.33–13.51; P.015). Interestingly, intraprocedural MAP decrease from its baseline value measured as absolute and percentage drop from its baseline value remained an independent risk factor of MBE occurrence in the cohort with complete reperfusion (TICI 3; aOR per 10 mmHg drop 1.38; 95% CI 1.01–1.89; P = 0.044, aOR per 10% fraction of baseline value drop 1.72; 95% CI 1.04–2.84; P = 0.036), but not in subgroups of only partial (TICI 2a and 2b) or unsuccessful reperfusion.

In the cohort of final TICI ≥ 2b, postprocedural SBP and MAP elevation were independent risk factors of sICH (aOR per 10 mmHg SBP increase 1.78; 95% CI 1.15–2.76; P = 0.010 and aOR per 10 mmHg MAP increase 1.78; 95% CI 1.04–3.03; P = 0.035).

Both sICH and MBE were associated with poor long-term functional outcomes and higher mortality (Table 1).

**Figure 1:**
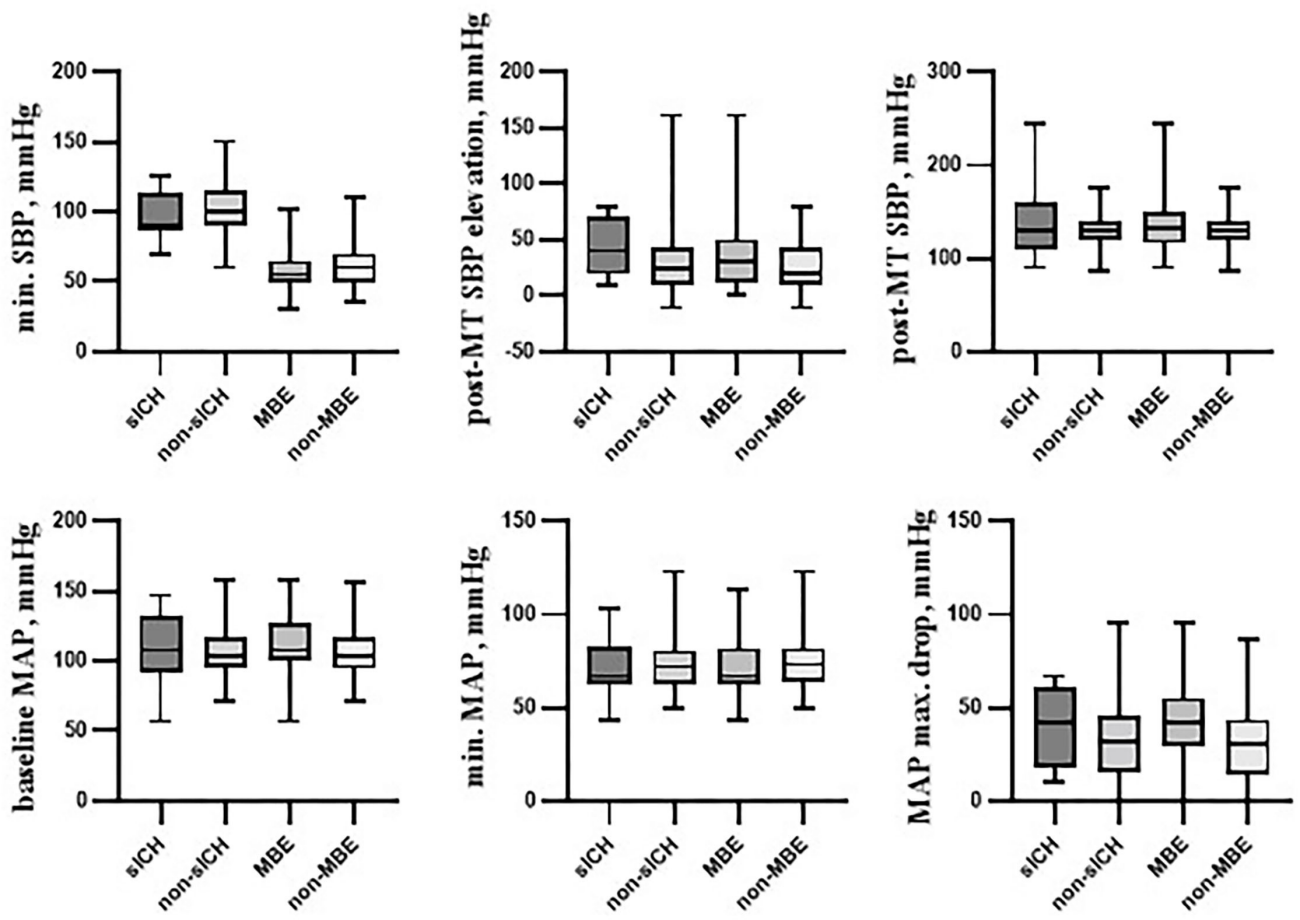
Selected hemodynamic variables according to primary outcome parameters. Box plots showing several hemodynamic variables in the following cohorts: individuals with symptomatic intracranial hemorrhage (sICH) and those without it (non-sICH), patients with malignant brain edema (MBE) and those without it (non-MBE). Among presented variables, post-mechanical thrombectomy systolic blood pressure elevation over its minimal intraprocedural value (post-MT SBP elevation) was found to be associated with the sICH occurrence. Variables associated with MBE occurrence were: baseline SBP, baseline systolic blood pressure; baseline MAP, baseline mean arterial blood pressure; MAP max. drop, post-MT SBP elevation, and maximal mean arterial blood pressure decrease from its baseline value. sICH, symptomatic intracranial hemorrhage; MBE, malignant brain edema; SBP, systolic blood pressure; MAP, mean arterial pressure; MT, mechanical thrombectomy; max., maximal; min., minimal.

Selected hemodynamic variables according to primary outcome parameters (sICH and MBE) were presented graphically in Figure 1.

## Discussion

We found that BP variations during MT treatment are associated with serious hemorrhagic and edematous early treatment complications. The intraprocedural MAP drop compared to its baseline value was an independent predictor of MBE and its over 40% decrement of both MBE and sICH occurrence. Postprocedural MAP elevation was associated with higher rates of sICH, and a rise in SBP with sICH and MBE incidence. The association of higher post-EVT BP values with those treatment complications stays in line with data from previous studies (7, 11).

Hemorrhagic transformation after EVT most probably results from the blood flow restoration in the settings of blood-brain-barrier (BBB) disruption and increased tissue permeability due to previous ischemic endothelial injury (5, 12). It corresponds well with the observation of similar risk factors for sICH, final infarct volume (FIV), and neurological deficit after reperfusion therapy (13, 14). According to this hypothesis, BP decreases during MT-treatment of AIS (prior to successful reperfusion or in case of only partial reperfusion) could lead to, not only enlargement of FIV but also endothelial ischemia and hemorrhagic complications, such as sICH (6). The risk of HT could then be further increased by BP elevation at the moment of recanalization or after successful recanalization, as was reported by some authors (7, 15). Our observation of SBP and MAP postprocedural elevation being predictors of sICH matches conclusions from those studies.

We found that large (>40%) MAP decreases during MT treatment are associated with higher rates of sICH in our entire cohort, but not in the subgroup with successful reperfusion. This may be explained by the observation that post-treatment TICI 2b-3 score significantly reduces the risk of sICH and it could potentially mitigate the hypoperfusion effect (16). Differences in the cerebral autoregulation impairment between complete (TICI 3) and incomplete (TICI 2a/2b) reperfusion might also influence the impact of intraprocedural hemodynamic changes on hemorrhagic treatment complications (17). Our study was not powered enough to adequately evaluate those associations. Given the theoretical considerations suggesting a relationship between systemic hypoperfusion and HT, the association between BP drops during MT and sICH deserves further evaluation in larger cohorts.

CED is a natural sequela of brain ischemia that primarily results from tissue necrosis (cytotoxic edema), but can also be the EVT complication secondary to the reperfusion syndrome or HT (5). Larger parenchymal hypoattenuation on CT, among others, was found to be a reliable predictor of its malignant course in the meta-analysis of studies that included non-selected patients with AIS (18). This confirms the link between higher infarct size and MBE occurrence. The association of MAP decreases during EVT and infarct volume growth may therefore suggest a similar relation between intraprocedural BP drops and MBE (6). This would be explained by the cytotoxic component of post-MT CED, being a result of larger FIV in patients with collateral flow compromised by the systemic hypotension. Our study results may confirm these considerations.

The weakness of our analysis is that we did not assess FIV. It is, therefore, impossible to differentiate between MBE being a result of larger infarct size or its other pathophysiological causes: reperfusion injury and HT. The finding of postprocedural SPB elevation being independently associated with MBE occurrence might suggest the role of reperfusion syndrome in its formation. However, this association did not reach statistical significance in the subgroup of patients with successful recanalization. The importance of reperfusion syndrome may be further underlined by the observation of several parameters of intraprocedural hypotension impact on MBE occurrence in the cohort of complete reperfusion, but not in the subgroups with only partial (TICI 2a and 2b) or unsuccessful reperfusion. This may suggest that patients with blood pressure decreases during MT treatment who achieved the treatment goal (full recovery of blood supply) may be at the greatest risk of MBE formation. Due to the limited number of included patients, our study probably does not have enough statistical power to fully evaluate those effects.

According to the literature, other factors that were found to be the post-EVT MBE predictors were unsuccessful recanalization, intracranial ICA occlusion, poor collateral status, and higher values of post-treatment BP (6, 18). BP values during and after treatment are the variables for which modification could potentially decrease the occurrence of this life-threatening complication (19). Considering the high rates of MBE after EVT [27% according to Huang et al. (20) 24% in our study], efforts should be made to further evaluate this problem.

Limitations of our study include the retrospective design and small sample size, with a potential for selection bias. Second, there was no protocol for periprocedural blood pressure maintenance that resulted in larger BP drops than those reported in other studies with a similar design (21). This might have potentiated the negative effect on outcome measures, but could also uncover associations that have not been shown earlier. Third, BP recorded every 5 min may only serve as a surrogate marker of continuous BP changes. Therefore, our results should be interpreted with caution. To our knowledge, we are the first to report the association of BP decreases during MT treatment with sICH and MBE.

In conclusion, MAP decrease during EVT compared to pre-treatment value is associated with MBE occurrence in the anterior circulation LVO cohort. Patients with large, over 40% MAP drop are at higher risk of sICH, but this effect is probably mitigated by the successful recanalization. Post-treatment elevation of SBP and MAP is the independent risk factor for sICH. Further evaluation of these effects is much needed to establish the optimal blood pressure targets in order to avoid serious treatment complications and improve outcomes.

## Data Availability Statement

The raw data supporting the conclusions of this article will be made available by the authors, without undue reservation.

## Ethics Statement

The studies involving human participants were reviewed and approved by Bioethics Committee of the University of Rzeszow. Written informed consent for participation was not required for this study in accordance with the national legislation and the institutional requirements.

## Author Contributions

MW designed the study, performed the analytic calculations, and wrote the manuscript. MS, KW, and HB-P collected data, discussed the results, and contributed to the final manuscript. All authors contributed to the article and approved the submitted version.

## Conflict of Interest

The authors declare that the research was conducted in the absence of any commercial or financial relationships that could be construed as a potential conflict of interest.

## Publisher's Note

All claims expressed in this article are solely those of the authors and do not necessarily represent those of their affiliated organizations, or those of the publisher, the editors and the reviewers. Any product that may be evaluated in this article, or claim that may be made by its manufacturer, is not guaranteed or endorsed by the publisher.
